# Low-Intensity Pulsed Ultrasound Promotes Osteogenic Potential of iPSC-Derived MSCs but Fails to Simplify the iPSC-EB-MSC Differentiation Process

**DOI:** 10.3389/fbioe.2022.841778

**Published:** 2022-05-12

**Authors:** Ziyi Hua, Shuang Li, Qianzi Liu, Minxuan Yu, Mengling Liao, Hongmei Zhang, Xuerong Xiang, Qingqing Wu

**Affiliations:** Chongqing Key Laboratory of Oral Diseases and Biomedical Sciences, Chongqing Municipal Key Laboratory of Oral Biomedical Engineering of Higher Education, Stomatological Hospital of Chongqing Medical University, Chongqing, China

**Keywords:** LIPUS, iPSCs, MSCs, EBs, osteogenic differentiation

## Abstract

Induced pluripotent stem cell (iPSC)-derived mesenchymal stem cells (iMSCs) are a promising cell source for bone tissue engineering. However, iMSCs have less osteogenic potential than BMSCs, and the classical iPSC-EB-iMSC process to derive iMSCs from iPSCs is too laborious as it involves multiple *in vitro* steps. Low-intensity pulsed ultrasound (LIPUS) is a safe therapeutic modality used to promote osteogenic differentiation of stem cells. Whether LIPUS can facilitate osteogenic differentiation of iMSCs and simplify the iPSC-EB-iMSC process is unknown. We stimulated iMSCs with LIPUS at different output intensities (20, 40, and 60 mW/cm^2^) and duty cycles (20, 50, and 80%). Results of ALP activity assay, osteogenic gene expression, and mineralization quantification demonstrated that LIPUS was able to promote osteogenic differentiation of iMSCs, and it worked best at the intensity of 40 mW/cm^2^ and the duty cycle of 50% (LIPUS40/50). The Wnt/β-catenin signaling pathway was involved in LIPUS40/50-mediated osteogenesis. When cranial bone defects were implanted with iMSCs, LIPUS40/50 stimulation resulted in a significant higher new bone filling rate (72.63 ± 17.04)% than the non-stimulated ones (34.85 ± 4.53)%. Daily exposure to LIPUS40/50 may accelerate embryoid body (EB)–MSC transition, but it failed to drive iPSCs or EB cells to an osteogenic lineage directly. This study is the first to demonstrate the pro-osteogenic effect of LIPUS on iMSCs. Although LIPUS40/50 failed to simplify the classical iPSC-EB-MSC differentiation process, our preliminary results suggest that LIPUS with a more suitable parameter set may achieve the goal. LIPUS is a promising method to establish an efficient model for iPSC application.

## Graphical Abstract

We demonstrate LIPUS at the intensity of 40mW/cm^2^ and the D.C of 50% (LIPUS40/50) can enhance the osteogenic potential of iMSCs in vitro and in vivo. LIPUS40/50 seems to accelerate EB-iMSC transition, but itfails to convert iPSCs or EB cells into osteoblasts directly. These results represent an important step toward applying iPSCs safely and effectively in bone tissue engineering.
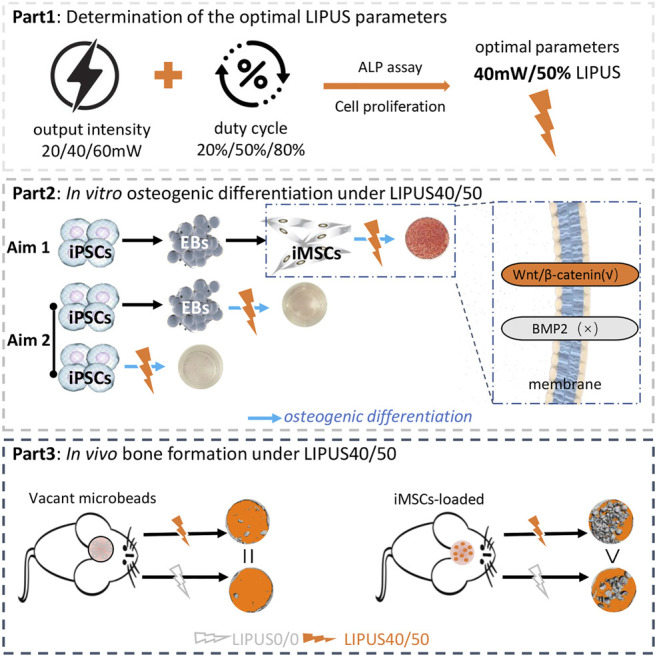

## 1 Introduction

Induced pluripotent stem cells (iPSCs), reprogrammed from somatic cells, are a special cell population with totipotency and multiple differentiation potential (Park et al., 2008; Li et al., 2009; Trokovic et al., 2014). In order to prevent tumorigenesis associated with totipotency, iPSCs were usually differentiated into MSC-like cells for application *in vivo*, named as iPSC-derived MSC-like cells (iMSCs) (Hynes et al., 2012; Pagni et al., 2012). However, two issues concerning the application of iPSCs remained: First, osteoblasts derived from iMSCs had less potential in osteogenic differentiation than those differentiated from bone marrow stromal cells (BMSCs), and the cells that failed to commit fully to osteogenic lineage might lead to immature bone formation and teratoma formation when transplanted back into bone defects (Duan et al., 2011; Jung et al., 2012; Diederichs and Tuan, 2014; Ko et al., 2014; Wang et al., 2015); second, the classical protocol of iMSC acquirement is laborious and inefficient, involving iPSC–embryoid body (EB) formation, iMSC harvest, subsequent MSC-like cell passage, and osteogenic differentiation (Hwang et al., 2008; Villa-Diaz et al., 2012; Tang et al., 2014; Wang et al., 2015). The two issues hindered the application iPSCs or iMSCs in bone tissue engineering, leaving it imperative to develop more efficient strategies for iMSC acquirement and osteogenic differentiation.

To promote the osteogenic efficiency of iPSC-derived cells, various attempts have been made, including scaffold modification and addition of chemical inducers, growth factors, and small molecules targeting certain pathways (Wu et al., 2017). All the strategies were designed to mimic the *in vivo* spatiotemporal signals during the osteogenic commitment by multipotent cells. Some procedures were reported to be capable of inducing iPSCs into osteogenic cells directly without EB formation (Chen et al., 2012; Phillips et al., 2014). However, it is hard to reproduce the complex and well-orchestrated differentiation scenarios by adding certain exogenous factors. Moreover, a bolus of exogenous osteogenic growth factors may lead to dystrophic mineralization due to non-physiologic osteoinduction (Wu et al., 2018). The application of small molecules targeting certain key pathways may be more precise. However, multiple molecules applied in a stepwise manner would be required to target the osteogenic switching points (Kanke et al., 2014), making the strategy too complex to be clinically translated. Therefore, the ideal strategy for iPSC application in bone tissue engineering is to awaken and amplify the host regenerative response, instead of adding certain chemical or biological exogenous factors.

Low-intensity pulsed ultrasound (LIPUS) below the intensity of 100 mW/cm^2^ is a safe and well-established therapeutic modality that is frequently used to accelerate fracture healing without surgical invasion (Pounder and Harrison, 2008; Loyola Sá nchez et al., 2009). The anabolic effect of LIPUS during bone healing was further evidenced by its positive effect on osteogenic differentiation of MSCs (Ebisawa et al., 2004; Sant'Anna et al., 2005; Schumann et al., 2006; Cui et al., 2007; Lai et al., 2010; Angle et al., 2011). Its osteogenic potential was reported to be associated with mechanical stress and increased expression of osteogenic growth factors activating osteogenic signaling pathways (Coords et al., 2011; Padilla et al., 2014). Particularly, studies proved that LIPUS can stimulate the expression of BMPs, which in turn commit themselves or neighboring cells to an osteogenic lineage in an autocrine or paracrine manner (Xue et al., 2013; Yang et al., 2014; Huang et al., 2015). All the aforementioned pieces of evidence indicate that LIPUS has the potential to awaken and amplify the endogenous osteogenic signals during natural bone regeneration.

Although BMSCs were reported to respond to LIPUS and commit to osteogenic lineage, it remains unknown whether LIPUS can facilitate the osteogenic differentiation of iMSCs and simplify the iPSC-EB-MSC differentiation process as iPSC-derived cells are less responsive to osteogenic inducers than BMSCs (Wang et al., 2015). We hypothesize that LIPUS can help realize the clinical application of iPSCs in bone tissue engineering by promoting osteogenic differentiation of iPSC-derived MSCs and/or simplifying the mesenchymal transition process of iPSCs. To test the hypothesis, two goals were set: 1) to figure out whether LIPUS can promote the osteogenic potential of iMSCs and 2) to study whether LIPUS can simplify the iPSC-EB-MSC differentiation process.

## 2 Materials and Methods

### 2.1 Derivation of iMSCs and Differentiation Assay

Human iPSC cell lines (ATCC^®^ ACS-1011™) reprogrammed from human foreskin fibroblasts were used in this study. After proliferation, iPSCs were induced into EBs by collecting sedimentation from iPSC clumps and resuspending in (EBs) culture medium composed of knockout DMEM (Invitrogen) supplemented with 20% knockout serum replacement (Invitrogen), 1 mM L-glutamine (Invitrogen), 1% non-essential amino acids (Invitrogen), 4 ng/ml basic fibroblast growth factor (Invitrogen), and 0.1 mM β-mercaptoethanol (Sigma) in ultra-low attachment cell culture flasks for 10 days. Then, EBs were seeded onto 0.1% gelatin-coated dishes in the MSC growth medium. Cells migrating out from the edge of EBs were subcultured until homogeneous fibroblastic morphology appeared. The cells differentiated from iPSCs are named iMSCs. The isolated EB cells and iMSCs were used for subsequent experiments. IMSCs were identified by fluorescence-activated cell sorting as in our previous study and multidirectional differentiation potential (Wu et al., 2018).

### 2.2 Effects of LIPUS on Cell Proliferation and Early Osteogenic Differentiation of iMSCs *In Vitro*

To test the effects of LIPUS on cell proliferation and early osteogenic differentiation, LIPUS at different intensities and duty cycle (DC) was an independent variable and the results of CCK-8 and ALP assays were dependent variables. LIPUS with different output intensities and DCs was generated by a LIPUS generation machine. The acoustic frequency was set at 1.5 MHz. Pulsed repetition frequency was set at 100 Hz. For analysis of cell proliferation, 2 × 10^5^ P4 iMSCs were seeded in each well of the six-well plates in MSC growth medium (*n* = 3). LIPUS reached the cells through a layer of coupling gel at the bottom of the six-well plate. One day after seeding, iMSCs were exposed to LIPUS at different parameters 10 min/day. As no data about the LIPUS parameter used for iPSC-derived cells were available, we set the output intensity at 20, 40, or 60 mW/cm^2^ and set the duty cycle (DC) at 20, 50, or 80%. Therefore, a total of nine experiment groups were carried out, namely, 20/20, 20/50, 20/80, 40/20, 40/50, 40/80, 60/20, 60/50, and 60/80 (output intensity/duty cycle). Cells cultured in the MSC growth medium without LIPUS stimulation were set as control. Cell proliferation was analyzed by a CCK-8 assay kit (Dojindo) after 1, 3, and 5 days of stimulation. For analysis of early osteogenic differentiation, 2 × 10^5^ P4 iMSCs were seeded in each well of the six-well plate in the osteogenic differentiation medium (ODM) to observe whether LIPUS and chemical inducers can work synergistically, or in the MSC growth medium to observe whether LIPUS alone can drive iMSCs to osteoblasts. ALP assay (Biovision) was carried out after 5 days of stimulation. The optimal parameters were defined as the output intensity and DC that can boost osteogenic differentiation of iMSCs while do not suppress cell proliferation significantly. The temperature of the LIPUS device is determined by infrared thermometer. All assays mentioned earlier were performed in triplicate.

### 2.3 Effects of the Optimal LIPUS on Late Osteogenic Differentiation of iMSCs *In Vitro*

The maturation and function of iMSC-derived osteoblasts with optimal LIPUS stimulation were further evaluated by q-PCR and mineralization assay. LIPUS at different intensities and DCs were independent variables, and the results of RT-PCR and mineralization assays were dependent variables. For control groups (*n* = 3), 2 × 10^5^ P4 iMSCs were seeded in each well of the six-well plates in the MSC growth medium until 90% confluence and replaced with the ODM without LIPUS stimulation. At 7 days and 14 days, total RNA was isolated from three wells using TRIzol reagent (TaKaRa). A constant amount of RNA (500 ng) of each sample was reverse-transcribed into cDNA by PrimeScript™ RT Master Mix (TaKaRa) using the following steps: 37°C for 15 min, 85°C for 5 s, 4°C. The expression of runt-related transcription factor 2 (Runx2), alpha-1 type 1 collagen (COL1A1), osteocalcin (OCN), and the housekeeping gene glyceraldehyde 3-phosphate dehydrogenase (GAPDH) was amplified and simultaneously quantified using SYBR^®^ Premix Ex Taq™ II (TaKaRa) under the following conditions: 95°C for 30 s, 95°C for 5 s, and 60°C for 60 s, 40 cycles.

Results were expressed relative to the gene expression level of iMSCs in the MSC growth medium without LIPUS stimulation on day 7 or 14, respectively. Xylenol orange (20 μM, Sigma) was added to the medium from day 7 to help monitor the calcium nodule formation in live osteogenic cell cultures. At day 21, the cultures were stained with Alizarin Red S, and quantification of mineralization was performed.

To explore the role of the BMP2 signaling pathway and Wnt/β-catenin signaling pathway in LIPUS-mediated osteogenic differentiation, a BMP2 antagonist (noggin, 200 ng/ml, R&D Systems) or Wnt/β-catenin pathway antagonist (Cardamonin, 10 μM, Sigma) was added to the osteogenic medium, followed by q-PCR, Alizarin Red staining, and quantification of mineralization. All assays mentioned before were performed in triplicate.

### 2.4 Effects of the Optimal LIPUS on Osteogenic Differentiation of iMSCs *In Vivo*

#### 2.4.1 Alginate Microbeads for Cell Delivery

Alginate microbeads were used as carriers for the cell delivery. Our previous study showed that the process of microencapsulation and subsequent injection had no adverse effects on the viability of encapsulated cells (Wu et al., 2018). Alginate microbeads were produced as reported earlier (Wu et al., 2018). Two groups of microbeads were produced: i. vacant microbeads (blank control) and ii. microbeads loaded with iMSCs: iMSCs were homogenized with a sterile alginate solution at a density of 2×10^6^ cells/mL. The alginate beads were washed three times with saline and then kept in DMEM for subsequent usage.

#### 2.4.2 *In Situ* Osteogenesis Model

The animal experiment was in accordance with guidelines established by the Institutional Animal Care and Committee of the Affiliated Stomatological Hospital of Chongqing Medical University. Mice with 5-mm calvarial bone defects were used. According to the grafted materials, the mice were divided into three groups for the *in vivo* study, namely, control (no microbeads), vacant microbeads, and iMSC-loaded microbeads. In each group, half of the animals received a LIPUS stimulation for 30 min every other day (*n* = 5), while the other half were stimulated with LIPUS0/0 as control (*n* = 5). LIPUS reached the skin and underlying tissues through a layer of coupling gel. Animals were kept in sterile microisolator cages under specific pathogen-free conditions.

In the *in vivo* study, the independent variables were LIPUS40/50 and loaded iMSCs, and the dependent variables were blood flow and bone filling rate. At 1 week, 4 weeks, and 12 weeks after surgery, blood flow within the bone defect was analyzed by infrared laser speckle imaging before killing (Pericam Perfusion Speckle Imager (PSI), Sweden). Under anesthesia, the skin overlying the defect was carefully elevated. A region of interest (ROI1) with a diameter of 5 mm was circled out by the margin of the defect. Another 5-mm ROI (ROI2) on the contralateral side on the normal bone surface was used as the calibrator for the measurement of sample’s blood flow (BF). The relative blood flow was determined by the ratio between ROI1 and ROI2. Samples were then harvested and scanned with micro-CT (Scanco Medical AG, mCT-80, Switzerland). The parameters were set at a voltage of 60 kV, an intensity of 142 mA, and an isotropic resolution of 7 μm. A threshold value of 220 mg HA/cm was set to distinguish mineralized tissue from the non-mineralized tissue (Man et al., 2012). Each sample was reconstructed, and the bone filling rate (%) was analyzed.

After scanning, all samples were decalcified, dehydrated, and embedded in paraffin. Serial sections were obtained for hematoxylin and eosin (H&E) staining and immunohistochemistry. The presence of antitumor necrosis factor α (TNF-α), anti-interleukin 10 (IL10), and the involvement of human-origin cells were detected using rabbit polyclonal anti-mouse TNF-α antibodies (1:50, Abcam), rat polyclonal anti-mouse IL10 antibodies (10 ug/ml, Abcam), and mouse monoclonal anti-human nuclei antibodies (1:50, Millipore), respectively. The slides were stained with primary antibodies at 4°C overnight and then incubated with secondary antibodies against rabbit, rat, or mouse IgG (1:1000, Abcam) for 30 min at 37°C. The immunohistological staining was revealed by streptavidin-HRP and diaminobenzidine substrates. The sections were counterstained with hematoxylin solution.

### 2.5 Potential of LIPUS in Simplifying the iPSC-EB-iMSC Differentiation Process

IPSCs were cultured in the growth medium or ODM. LIPUS40/50 was applied to iPSCs to observe the effects of LIPUS on clone formation and direct osteogenic differentiation of iPSCs. At 16 days, the osteogenic cultures were stained with Alizarin Red S, and quantification of mineralization was performed. EBs were formed from iPSCs and seeded in the EB-MSC differentiation medium. LIPUS40/50 was applied to EBs to observe the effects of LIPUS on EB-iMSC conversion. EB cells were dissociated and seeded in the ODM. LIPUS40/50 was applied to the EB cells to observe the effects of LIPUS on direct osteogenic differentiation of EB cells. All assays mentioned earlier were performed in triplicate.

### 2.6 Statistical Analyses

Statistical analyses were performed using SPSS 19.0. All data were expressed as the mean value ±standard deviation (SD). The Levene test was first performed to check the normality of the distribution and if equal variance assumption of the data was violated or not. One-way analysis of variance (ANOVA) was used to analyze the mineral quantification among multiple groups. The Student–Newman–Keuls test was applied as the post hoc test after ANOVA. The Student t test was used to analyze the difference between each experimental group and the control at the same time point, regarding the data of cell proliferation, ALP activity, fold changes of gene expression, blood flow, and micro-CT results. A confidence level of 95% (*p* < 0.05) was considered significant.

## 3 Results

### 3.1 Effects of LIPUS on Cell Proliferation and Early Osteogenic Differentiation of iMSCs *In Vitro*

**FIGURE 1:**
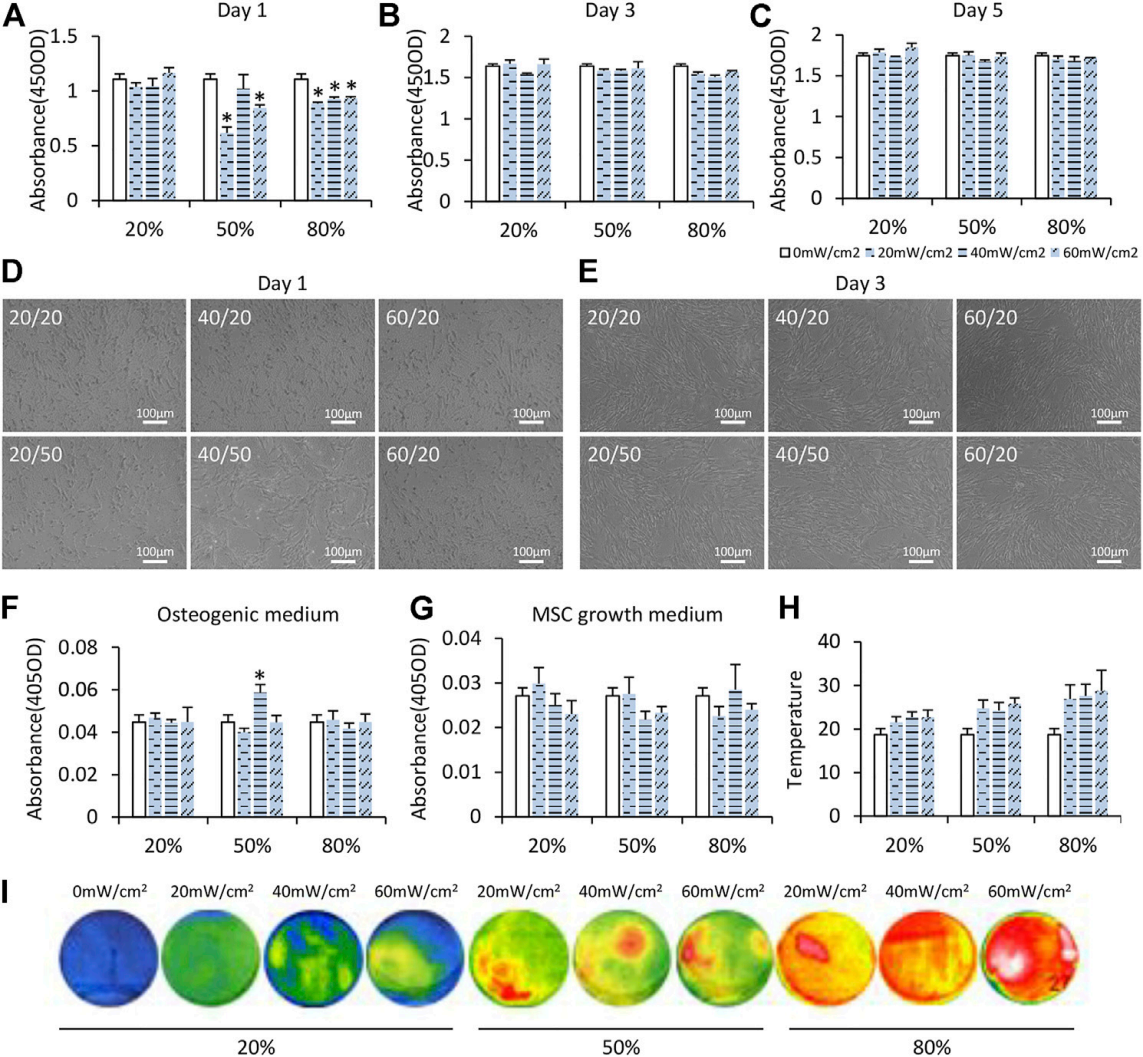
Preliminary selection of the optimal LIPUS parameter for iMSC differentiation. **(A–C)** CCK-8 assay of iMSCs under daily LIPUS stimulation on days 1, 3, and 5. Different output intensities and duty cycles were tested. **(D,E)** IMSCs under LIPUS stimulation on days 1 and 3. **(F,G)** ALP activity was evaluated on day 5. Cells were cultured in the osteogenic medium or MSC growth medium. **(H,I)** Temperature of the LIPUS device increased with DC determined by an infrared thermometer.

LIPUS with nine sets of parameters was used to test the effects of LIPUS on iMSCs preliminarily. Significant changes in cell proliferation rate were found on day 1 when iMSCs were exposed to LIPUS but not on day 3 or day 5 (Figures 1A–C). When the DC was 20%, cell proliferation at 20 mW/cm^2^, 40 mW/cm^2^, and 60 mW/cm^2^ was not significantly different from that at 0 mW/cm^2^ (Figure 1A). When the DC was 50%, cell proliferation was significantly suppressed at 20 mW/cm^2^ and 60 mW/cm^2^ but not at 40 mW/cm^2^ (Figure 1A). When DC was 80%, cell proliferation was suppressed at all varied output intensities (Figure 1A). The suppressive effect of LIPUS on cell proliferation was transient as no significant changes were found when iMSCs were cultured for more than 3 days (Figures 1B,C). The shape and layout pattern of iMSCs were observed on day 1 and day 3. On day 1, iMSCs under the stimulation of LIPUS40/50 were more fibroblast-like and interconnected than other groups (Figure 1D). On day 3, all groups reached 80% confluence, and no obvious changes were found in cell morphology and layout (Figure 1E).

To observe early osteogenic differentiation of iMSCs under LIPUS, iMSCs were cultured in the ODM and stimulated with LIPUS for 5 days. Only LIPUS40/50 had a synergistic effect with the chemical inducers in ODM as revealed by the ALP activity assay (Figure 1F). Without chemical inducers, LIPUS alone showed no significant effects on the osteogenic potential of iMSCs (Figure 1G). The surface temperature of the LIPUS device increased with DC (Figures 1H,I). The highest temperature was about 27°C when the DC was 80%. When the DC was 50%, similar temperature was observed at 20 mW/cm^2^, 40 mW/cm^2^, and 60 mW/cm^2^, suggesting that the effect of LIPUS40/50 on cell morphology and early osteogenic potential was not attributable to the thermal effect. The optimal parameters of LIPUS were defined as the output intensity and DC that can boost osteogenic differentiation of iMSCs while do not suppress cell proliferation significantly. The results of cell proliferation, cell morphology, and early osteogenic differentiation mentioned before indicated that the optimal parameters for osteogenic differentiation of iMSCs were possibly 40/50.

### 3.2 Effects of the Optimal LIPUS on Late Osteogenic Differentiation of iMSCs *In Vitro*

**FIGURE 2:**
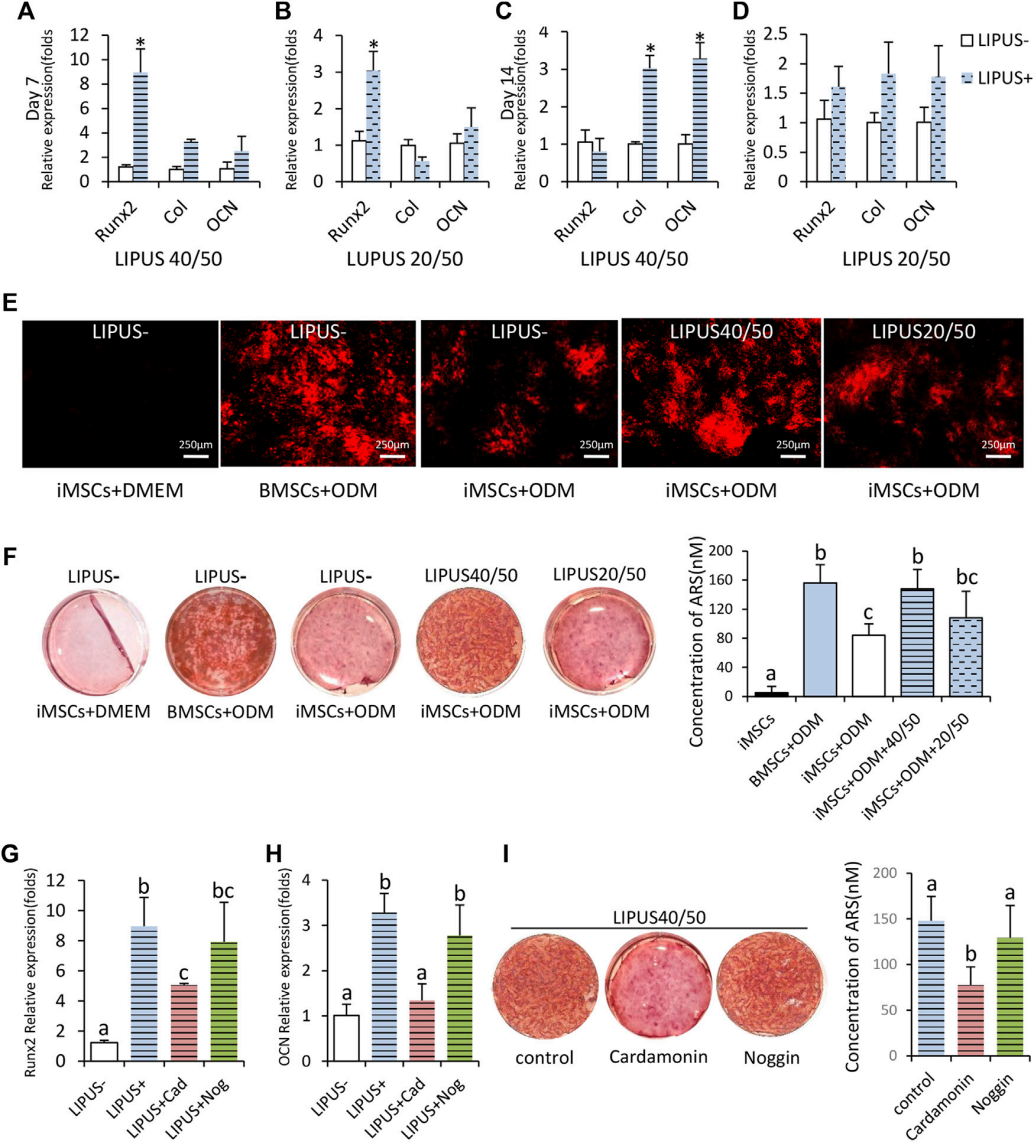
Osteogenic potential of iMSCs under the stimulation of LIPUS40/50 *in vitro*. **(A–D)** Fold changes in the expression level of Runx2, Col, and OCN. IMSCs underwent osteogenic differentiation with/without the LIPUS stimulation, *n* = 3. Data are presented as mean ± SD, *p<0.05. **(E)** Xylenol orange staining; LIPUS40/50 greatly increased the positive staining area. **(F,I)** Alizarin Red staining and mineralization quantification. Data are presented as mean ± SD, *n* = 3. Bars with dissimilar letters indicated significantly different values (*p* < 0.05). **(G,H)** Fold changes in the expression level of Runx2 and OCN, *n* = 3. Bars with dissimilar letters indicated significantly different values (*p* < 0.05).

To further test the pro-osteogenic potential of LIPUS40/50, osteogenic assay was performed with longer exposure time. To prove that 40/50 was the optimal, iMSCs were also stimulated with parameters 20/50 as a non-optimal LIPUS control. On day 7, expression of Runx2 increased at both 40/50 and 20/50, but the difference was more notable at 40/50 (Figures 2A,B). The expression of COL1A1 on day 7 significantly increased under the stimulation of LIPUS40/50 but not LIPUS20/50, indicating LIPUS40/50 accelerated expression of early and mid-stage osteogenic genes (Figures 2A,B). On day 14, the expression of COL1A1 and OCN increased significantly at 40/50 (*p* < 0.05), while only a slight increase was observed at 20/50 (*p* > 0.05) (Figures 2C,D).

Xylenol orange staining was used for real-time detection of mineral deposition. In the culture of BMSCs and iMSCs in the ODM, red fluorescent spots, the early markers of mineral deposition, were first detected on days 9 and 11, respectively. Under the stimulation of LIPUS40/50 and LIPUS20/50, mineral deposition was first detected on days 8 and 11, respectively, proving that LIPUS40/50 accelerated osteogenic differentiation. On day 16, the staining plaques in BMSCs + ODM culture were more prominent than those in iMSCs + ODM culture, indicating iMSCs owned a lower osteogenic potential than BMSCs (Figure 2E). When exposed to LIPUS, positive staining areas in iMSC culture were greatly increased at 40/50 compared with the LIPUS− control (Figure 2E). LIPUS20/50 displayed a much weaker potential to increase mineral deposition than LIPUS40/50 (Figure 2E). On day 21, mineralization in iMSCs + ODM culture almost doubled when exposed to LIPUS40/50 (*p* < 0.05), reaching a level comparable to that in the BMSCs + ODM group (Figure 2F). LIPUS20/50 showed a lower potential to promote mineralization than LIPUS40/50 (Figure 2F). In generation, LIPUS40/50 showed a significant pro-osteogenic effect on iMSCs, which was selected as the optimal parameter used in the following studies.

To uncover the molecular mechanism behind the pro-osteogenic effect of LIPUS40/50, antagonists of the Wnt/β-catenin signaling pathway or BMP2 signaling pathway was added to the osteogenic medium. The elevation in expression of Runx2 and OCN stimulated by LIPUS40/50 were suppressed by cardamonin, rather than noggin (Figures 2G,H). Mineral quantification further revealed that cardamonin but not noggin significantly decreased the mineral nodule formation compared with the control (Figure 2I), indicating the pro-osteogenic effect of LIPUS40/50 was related to the Wnt/β-catenin signaling pathway.

### 3.3 Effects of the Optimal LIPUS on Osteogenic Differentiation of iMSCs *In Vivo*

**FIGURE 3:**
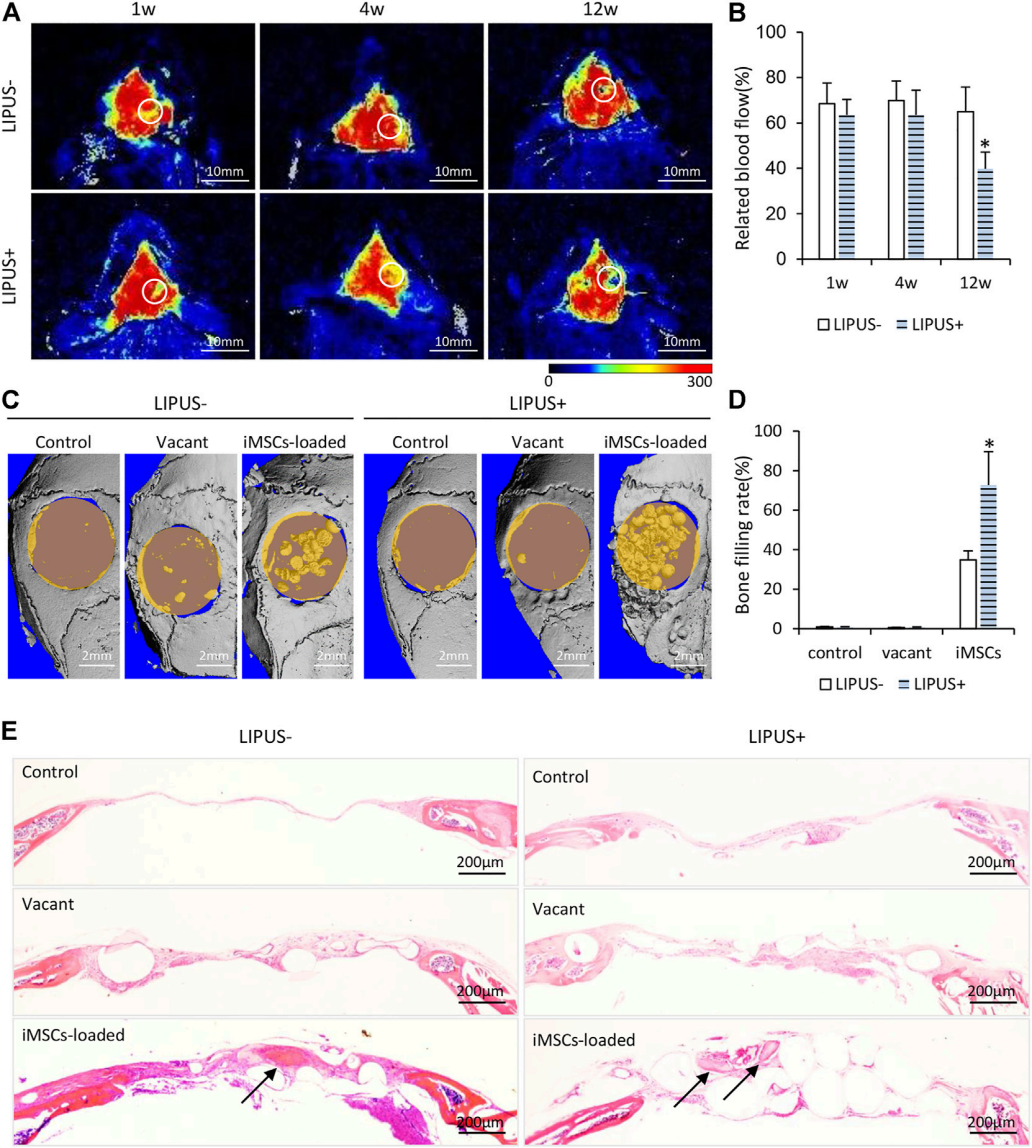
Osteogenic potential of iMSCs under the stimulation of LIPUS40/50 *in vivo*. **(A)** Infrared laser speckle imaging of the bone defect implanted of iMSC-loaded microbeads with/without LIPUS40/50 at 1, 4, and 12 weeks. The color scale showed the blood flow from 0 (dark blue) to 300 ml min^−1.^100 g^−1^ tissue (red). ROIs were determined by the contour of the bone defect. **(B)** Blood flow quantification at 1, 4, and 12 weeks; *n* = 5. **(C)** Micro-CT reconstruction of bone defect implanted with empty microbeads or iMSC-loaded microbeads. Bone defect without implants as control. **(D)** Bone filling rate was evaluated by micro-CT reconstruction. **(E)** HE staining images at 12 weeks after surgery. Arrows indicate new bone formation.

To study the *in vivo* effects of LIPUS, we further implanted microbeads encapsulated with iMSCs in calvarial bone defects. Material implantation caused no signs of rejection, infection, or tissue necrosis throughout the entire inspection period. The mapping of blood flow velocity by laser speckle flowmetry is displayed in Figure 3A. At 1 week and 4 weeks, the defects implanted with iMSC-loaded microbeads exhibited comparable blood flow in the LIPUS− and the LIPUS+ group (Figures 3A,B). The stimulation of LIPUS40/50 resulted in a lower blood flow than the LIPUS− control at 12 weeks (*p* < 0.05) (Figures 3A,B), demonstrating LIPUS is incapable of increasing blood supply.

According to the micro-CT reconstruction, almost no new bone formation was found in the control or the vacant microbead group, with or without LIPUS stimulation, indicating LIPUS alone has no pro-osteogenic potential in critical bone defects (Figures 3C,D). When iMSCs were loaded in the microbeads, obvious new bone formation was found in the center and along the margin of the defect, leading to a significant increase in bone filling rate compared with the control and vacant microbead group (Figures 3C,D). When the iMSC-loaded microbeads were stimulated with LIPUS40/50, the bone filling rate (72.63 ± 17.04%) was further elevated to be statistically higher than that without LIPUS stimulation (34.85 ± 4.53%) (Figures 3C,D). In the control and vacant microbead group, HE staining demonstrated neither new bone formation nor microbead mineralization, whether with LIPUS stimulation or not (Figure 3E). A larger population of inflammatory cells was observed in the LIPUS-stimulated defect than in the non-stimulated one (Figure 3E). In the iMSC-loaded microbead group, inter-microbead new bone formation and microbead mineralization were found in the defects (Figure 3E). LIPUS stimulation resulted in a larger amount of iMSC-loaded microbead mineralization, suggesting LIPUS may stimulate the production and release of osteogenic factors from the implanted iMSCs.

**FIGURE 4:**
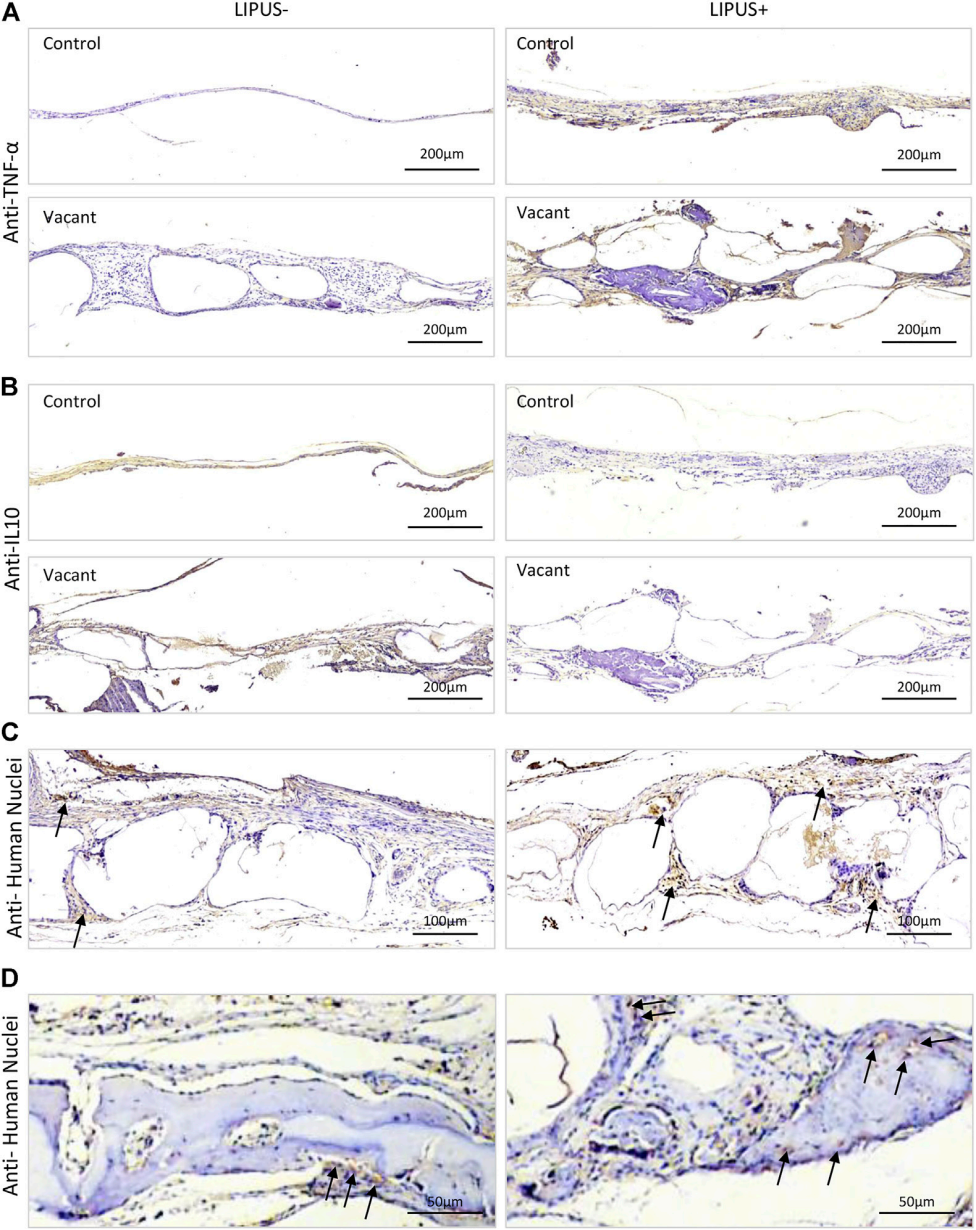
Representative IHC staining images after 12 weeks of LIPUS stimulation *in vivo*. **(A)** IHC staining for TNF-α. **(B)** IHC staining for IL10. **(C,D)** IHC staining for human nuclei. Black arrows represent positively stained human cells.

To figure out whether LIPUS stimulated inflammation, TNF-α and IL10 immunohistochemistry were performed. Strong/positive staining against TNF-α and week/negative staining against IL10 were observed in the control and vacant groups under daily LIPUS stimulation, while weak/negative staining against TNF-α and strong/positive staining against IL10 were observed in those without LIPUS stimulation (Figures 4A,B). The stronger staining of TNF-α and weaker staining of IL10 due to LIPUS40/50 stimulation indicated that LIPUS had a pro-inflammatory effect, although a significantly higher amount of new bone formation was found in the stimulated defects. IHC staining for human nuclei (MAB) was conducted to show the participation of loaded iMSCs in new bone formation (Figure 4C). Sections under LIPUS stimulation displayed many MAB-positive cells in the inter-microbead soft tissue and new bone, while those with no LIPUS stimulation showed only a few positive cells in the soft tissue (Figure 4C), suggesting LIPUS facilitates the migration of iMSCs from the microbeads to the inter-microbead space.

### 3.4 Potential of LIPUS in Simplifying the iPSC-EB-iMSC Differentiation Process

**FIGURE 5:**
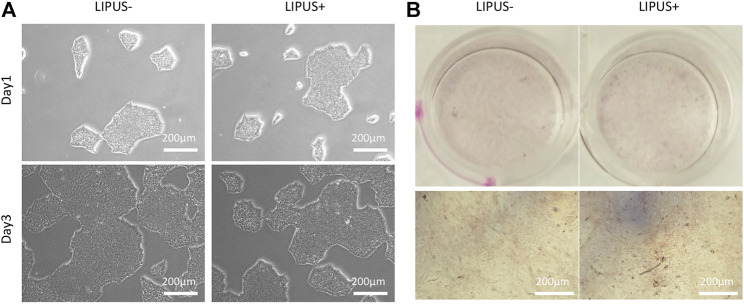
Effects of LIPUS40/50 on clone formation **(A)** and direct osteogenic differentiation of iPSCs **(B)**.

LIPUS40/50 was used to stimulate iPSCs or EB cells to observe its effects on cell differentiation. In the culture of iPSCs, typical iPSC clones were displayed and no MSC-like cells were found under LIPUS stimulation (Figure 5A). The cultures reached >80% confluence on day 3 with or without LIPUS stimulation (Figure 5A). When cultured directly in the ODM, the chemical inducers failed to produce mineralized nodule, whether with LIPUS stimulation or not (Figure 5B).

**FIGURE 6:**
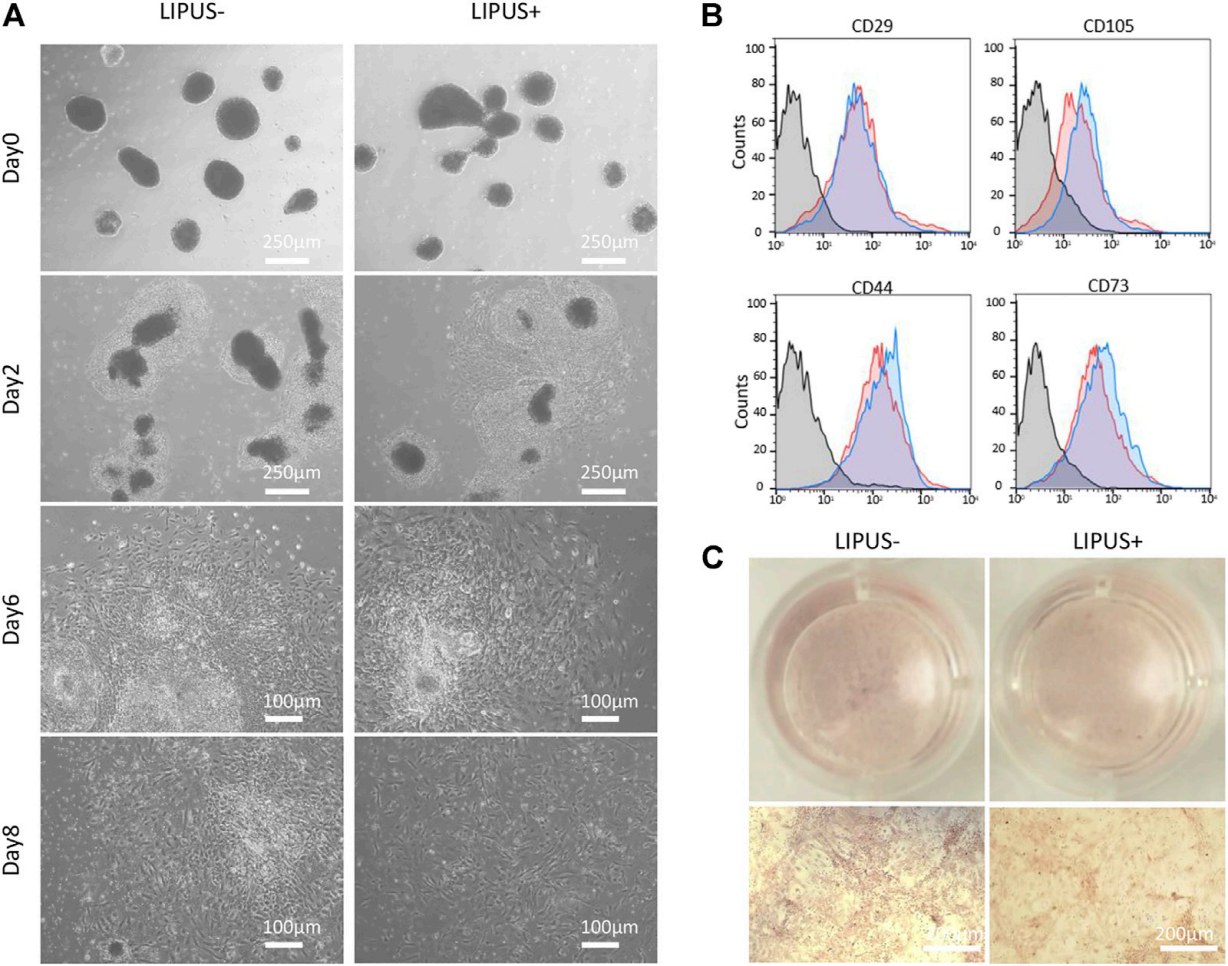
Effects of LIPUS40/50 on EB-MSC differentiation and direct osteogenic differentiation of EB cells. **(A)** Migration of MSC-like cells from EBs with/without LIPUS40/50 on days 0, 2, 6, and 8. **(B)** Phenotype of P2 iMSCs produced from EBs with/without LIPUS40/50. CD29, CD105, CD44, and CD73 were typical mesenchymal positive markers. **(C)** Alizarin Red staining of dissociated EB cells cultured in the ODM with/without LIPUS40/50.

The iMSC differentiation from EB cells tends to be accelerated by LIPUS stimulation (Figure 6A). After 2 days of EB seeding, the EB cells were surrounded by a condense band of MSC progenitor cells in both the LIPUS− and LIPUS+ groups, while a moderate number of MSC-like cells were found migrating out of the progenitor band in the LIPUS+ group (Figure 6A). After 6 days of EB seeding, both groups displayed a large number of MSC-like cells, while the cells under LIPUS stimulation were larger and more elongated (Figure 6A). Almost no condensed progenitor cell clone was found in the LIPUS+ group on day 8, while a few remained in the LIPUS− group (Figure 6A). The iMSCs from both groups were passaged and then underwent flowmetry. The LIPUS+ group tends to show a more mesenchymal phenotype (Figure 6B). Dissociated EB cells were directly cultured under osteogenic medium, and the chemical inducers failed to produce the mineralized nodule whether with LIPUS stimulation or not (Figure 6C). Those results indicated that LIPUS may accelerate the mesenchymal differentiation of EB cells, but it failed to transform iPSCs or EBs into osteogenic cells directly, demonstrating that LIPUS40/50 is incapable of simplifying the classical iPSC-EB-iMSC differentiation process.

## 4 Discussion

IPSC is a promising cell source for bone regenerative medicine. However, the clinical application of IPSC-derived cells was hindered by their inadequate osteogenic potential and laborious acquirement process. It remains a challenge to determine an efficient and safe conversion of iPSCs into osteogenic cells. LIPUS is proven to be a convenient and non-invasive treatment on bone regeneration (Takayama et al., 2007). In this study, we proved that LIPUS at an intensity of 40 mW/cm^2^ and a DC of 50% can promote the osteogenic potential of iMSCs. Although it failed to simplify the classical iPSC-EB-MSC differentiation process, LIPUS40/50 may accelerate iMSCs’ migration out of EBs. In generation, LIPUS is a promising strategy to help achieve clinical application of iPSCs.

Cell proliferation was a prerequisite for the success of osteogenesis by cells implanted in a bone defect. The effects of LIPUS on cell proliferation were studied in the first place to choose the optimal parameters for iMSC culture and differentiation. The effect of LIPUS on cell proliferation remains debatable in previous studies. On the one hand, Takayama et al. reported that transient LIPUS stimulation did not affect the proliferation rate of ROS 17/2.8 cells (Takayama et al., 2007). Parvizi et al. reported that pulsed ultrasound had no effects on rat chondrocyte proliferation (Parvizi et al., 1999). On the other hand, Zhang et al. reported that LIPUS influenced chondrocyte proliferation in an intensity- and a DC-dependent manner (Zhang et al., 2003). LIPUS does not promote or may even inhibit cell proliferation as the intensity and DC of LIPUS continue to decrease or increase excessively (Welgus et al., 1981; Chang et al., 2002). In this study, we found that LIPUS led to no increase in cell proliferation at any of the nine sets of parameters. In fact, LIPUS had a transient suppressive effect on iMSC proliferation on the first day of stimulation when the DC was 50 and 80%. Suppression may be related to thermal effects as indicated by the higher temperature at DC 50 and 80% than at 20%. However, suppression was not detected in the LIPUS40/50 group, although LIPUS20/50, LIPUS40/50, and LIPUS80/50 displayed similar temperature. This observation indicated that LIPUS40/50 may drive an early onset of certain mechanisms that offset the suppressive effects on proliferation. When iMSCs were exposed to LIPUS for more than 3 days, its effects on cell proliferation vanished, suggesting these parameters were safe and the differences in their osteogenic potential were not attributed to changes in cell proliferation.

The expected pro-osteogenic effect of LIPUS on iMSCs was achieved in this study, especially at 40/50. The ODM containing chemical inducers like ascorbic acid, β-glycerophosphate, and dexamethasone is the most commonly used chemical formulation for osteogenic differentiation of iPSC-MSCs (TheinHan et al., 2013; Diederichs and Tuan, 2014; Ishiy et al., 2015). However, studies revealed that the iPSC-derived cells were less osteogenic potential than BMSCs under the induction of chemical cues alone (Diederichs and Tuan, 2014; Ko et al., 2014; Wang et al., 2015). The fact that LIPUS worked synergistically with chemical inducers has been reported by previous studies using osteogenic cells other than iPSC-derived cells. LIPUS at an intensity of 30 mW/cm^2^, and a DC of 20% can significantly promote the osteogenic or chondrogenic differentiation of BMSCs (Cheung et al., 2013; Kusuyama et al., 2014; Yamaguchi et al., 2016). LIPUS at an intensity of 100 mW/cm^2^ and a DC of 20% has a significant positive effect on osteogenic differentiation of adipose-derived stem cells (Jiang et al., 2012; Yue et al., 2013). Mirzaei et al. also reported that physical stimulation like extremely low-frequency pulsed electromagnetic field worked synergistically with PVDF-polyaniline to amplify the osteogenic differentiation ability of stem cells (Mirzaei et al., 2019). Given that iMSCs have less osteogenic potential than BMSCs, it was expected that the optimal parameters at which LIPUS works with iMSCs may be different from those reported by studies on BMSCs. Our study found that the osteogenic differentiation of iMSCs was significantly upregulated by LIPUS at an intensity of 40 mW/cm^2^ and DC of 50%. The DC seems to play a crucial role here as DC of 20% or 80% had no pro-osteogenic potential on iMSCs even when intensity increased. However, LIPUS has no obvious pro-osteogenic effects if iMSCs were induced in the MSC growth medium, suggesting LIPUS can only work synergistically with chemical inducers.

How LIPUS enhances the osteogenic differentiation of iMSCs remains unclear. It was reported that LIPUS can promote expression and release of various osteogenic growth factors in cells, such as BMP2 (Padilla et al., 2014; Harrison et al., 2016; An et al., 2018). The activation of the Wnt/β-catenin signaling pathway was reported to be involved in the pro-osteogenic effect of LIPUS on BMSCs (Li et al., 2018). Our study preliminarily tested the molecular mechanisms and found that the Wnt/β-catenin pathway, but not the BMP2 pathway, was associated with the pro-osteoclastogenic effect of LIPUS on iMSCs. How LIPUS promoted bone regeneration *in vivo* was more complex to explain. Blood supply and early immune response were important *in vivo* factors that determined new bone formation (Scadden, 2016; Cheng et al., 2021). Previous studies have shown that LIPUS could promote angiogenesis (Zhu et al., 2015; Kang et al., 2019) and suppress inflammation (Nakamura et al., 2011; Chung et al., 2012; Lv et al., 2015). In our study, however, LIPUS40/50 showed no potential in increasing blood supply and suppressing inflammation. The conflicting results may be related to the different parameters and experimental methods. Based on the fact that LIPUS induced almost no new bone formation when iMSCs were not loaded, and LIPUS promoted the direct participation of iMSCs in tissue regeneration, it was suggested that the pro-osteogenic potential of LIPUS *in vivo* should be largely attributed to the direct effects of LIPUS on the transplanted cells.

To figure out whether LIPUS can simplify the iMSC production process, we cultured iPSCs or EB cells directly in the ODM and stimulated the culture with LIPUS. It turned out that LIPUS40/50 was incapable of inducing osteogenic differentiation of iPSCs or dissociated EB cells, suggesting LIPUS40/50 failed to ignite the signaling pathway associated with mesenchymal differentiation. However, LIPUS40/50 did seem to promote the migration of MSC-like cells from the EBs, and the stimulated P2 MSC-like cells showed a more mesenchymal phenotype. We assume that the optimal parameters for osteogenic differentiation of iMSCs, EBs, and iPSCs may be different. Future studies are necessary to replicate the osteogenic test using LIPUS of varied parameters.

This study has a few limitations. As the *in vivo* study revealed pro-inflammatory responses induced by LIPUS40/50, *in vitro* inflammation assays should be performed in future studies to unravel the mechanisms of LIPUS in promoting bone regeneration. LIPUS40/50 was applied in the direct differentiation assay of iPSCs and EBs, which was the optimal parameter selected by the osteogenic assay of iMSCs. Future studies are needed to test other parameters. At last, the somatic source of the iPSCs used in this study was a foreskin fibroblast cell line, rather than bone cells, which may own a lower osteogenic potential and interfere with the LIPUS function. iPSCs from other somatic sources should also be tested to reveal the pro-osteogenic potential of LIPUS.

## 5 Conclusion

In this study, we not only demonstrated for the first time the significant effect of LIPUS on the osteogenic differentiation of iMSCs but also figure out the optimal parameter that may guide iMSCs toward mature osteogenic phenotypes. Although LIPUS at an intensity of 40 mW/cm^2^ and a DC of 50% failed to simplify the classical iPSC-EB-MSC differentiation process, the preliminary results suggest LIPUS with a more suitable parameter set may achieve the goal. The combined application of LIPUS and iPSC-derived cells may become a promising alternative for the treatment of large bone defects.

## Data Availability

The original contributions presented in the study are included in the article/Supplementary Material, further inquiries can be directed to the corresponding author.
